# Directed Evolution Generates a Novel Oncolytic Virus for the Treatment of Colon Cancer

**DOI:** 10.1371/journal.pone.0002409

**Published:** 2008-06-18

**Authors:** Irene Kuhn, Paul Harden, Maxine Bauzon, Cecile Chartier, Julie Nye, Steve Thorne, Tony Reid, Shaoheng Ni, Andre Lieber, Kerry Fisher, Len Seymour, Gabor M. Rubanyi, Richard N. Harkins, Terry W. Hermiston

**Affiliations:** 1 Novel Technologies, Bayer Healthcare, Richmond, California, United States of America; 2 Palo Alto Veteran's Hospital and Stanford University, Palo Alto, California, United States of America; 3 Division of Medical Genetics, Department of Medicine, University of Washington, Seattle, Washington, United States of America; 4 Hybrid systems Ltd, Oxfordshire, United Kingdom; University of Hong Kong, China

## Abstract

**Background:**

Viral-mediated oncolysis is a novel cancer therapeutic approach with the potential to be more effective and less toxic than current therapies due to the agents selective growth and amplification in tumor cells. To date, these agents have been highly safe in patients but have generally fallen short of their expected therapeutic value as monotherapies. Consequently, new approaches to generating highly potent oncolytic viruses are needed. To address this need, we developed a new method that we term “Directed Evolution” for creating highly potent oncolytic viruses.

**Methodology/Principal Findings:**

Taking the “Directed Evolution” approach, viral diversity was increased by pooling an array of serotypes, then passaging the pools under conditions that invite recombination between serotypes. These highly diverse viral pools were then placed under stringent directed selection to generate and identify highly potent agents. ColoAd1, a complex Ad3/Ad11p chimeric virus, was the initial oncolytic virus derived by this novel methodology. ColoAd1, the first described non-Ad5-based oncolytic Ad, is 2–3 logs more potent and selective than the parent serotypes or the most clinically advanced oncolytic Ad, ONYX-015, *in vitro*. ColoAd1's efficacy was further tested *in vivo* in a colon cancer liver metastasis xenograft model following intravenous injection and its *ex vivo* selectivity was demonstrated on surgically-derived human colorectal tumor tissues. Lastly, we demonstrated the ability to arm ColoAd1 with an exogenous gene establishing the potential to impact the treatment of cancer on multiple levels from a single agent.

**Conclusions/Significance:**

Using the “Directed Evolution” methodology, we have generated ColoAd1, a novel chimeric oncolytic virus. *In vitro,* this virus demonstrated a >2 log increase in both potency and selectivity when compared to ONYX-015 on colon cancer cells. These results were further supported by *in vivo* and *ex vivo* studies. Furthermore, these results have validated this methodology as a new general approach for deriving clinically-relevant, highly potent anti-cancer virotherapies.

## Introduction

The development of effective treatments for human solid tumors remains a significant challenge to cancer researchers and oncologist alike. This is due to the complexity of human solid tumors, with multiple, sometimes redundant, interacting signaling pathways [1], patient population differences [2], and the ability to acquire resistance to treatments including the newly developed targeted molecular therapies such as erlotinib, gefitinib, and imatinib [3]. Consequently, new agents, with unique mechanisms of action capable of confronting this complexity, are needed.

Oncolytic viruses are unique anti-cancer agents capable of amplifying the input dose through replication in a tumor-dependent fashion. Human adenovirus (Ad) is one of a series of viruses being developed as oncolytic agents to treat human malignancies [4]. Early clinical trials and pre-clinical studies have demonstrated synergy of this type of novel cancer therapy with standard of care chemotherapy [5] and radiation [6], [7], [8]. However, while the oncolytic Ads tested in clinical trials have demonstrated marked safety, they have shown limited clinical efficacy as monotherapies [9], [10], [11], [12]. Consequently, several approaches are being explored to increase their potency (defined as the viruses ability to replicate, lyse cells, and spread), including increasing the efficiency of cell lysis [13], [14], [15], [16], [17], [18], infectivity [19], and “arming” them with therapeutic transgenes [20].

There are 51 defined human Ad serotypes, grouped A–F and these serotypes differ at a variety of levels (e.g. pathology in humans and rodents, hemagglutinatin properties, cellular receptors). However, with the exception of fiber alterations [19], alternative human Ad serotypes to the well studied Ad5 serotype have been ignored. Thus alternative serotypes may represent an unexplored avenue for developing more potent virotherapies.

To fully explore their potential, we employed a methodology we term “Directed Evolution”, in which pools of Ad serotypes, representing the different Ad subgroups, are passaged on human tumor cell lines representative of major solid tumor indications (breast, colon, pancreatic, prostate) to invite recombination and selection of potent viral variants or serotypes. This simple, non-prejudiced approach utilizes the complexity of the human tumor cell to direct the evolution of select, highly potent Ads from the pool and is very appealing since it can be directed toward an outcome (e.g. developing a more lytic virus) without prejudice towards the mechanism(s) that may be responsible for that outcome (e.g. efficiency of cell lysis, infectivity, viral DNA replication). ColoAd1, a virus isolated from the colon cell line-passaged viral pool, displayed potency superior to Ad5 on a series of colon tumor cell lines, and a wider therapeutic window on a collection of colon tumor lines and primary normal cells than the recently approved and marketed virotherapy, ONYX-015/H101[21]. The superior potency was further demonstrated *in vivo* in a liver tumor seeding model and the selectivity was validated on clinically excised colon cancer tissue. In addition, we demonstrate that we can “arm” this novel agent by incorporating transgenes into the viral genome without compromising potency of the agent, thus increasing the potential of this agent to deal with the complexity of human solid tumors. This is the first description of a non-Ad5 based oncolytic Ad, and the exploitation of alternative Ad serotypes marks a novel approach to the development of more potent and selective oncolytic viruses for the treatment of human cancers.

## Results

### Directed Evolution of pooled adenovirus serotypes on different tumor cell lines derives distinctly different viral pools that are superior in potency to Ad5

**Figure 1:**
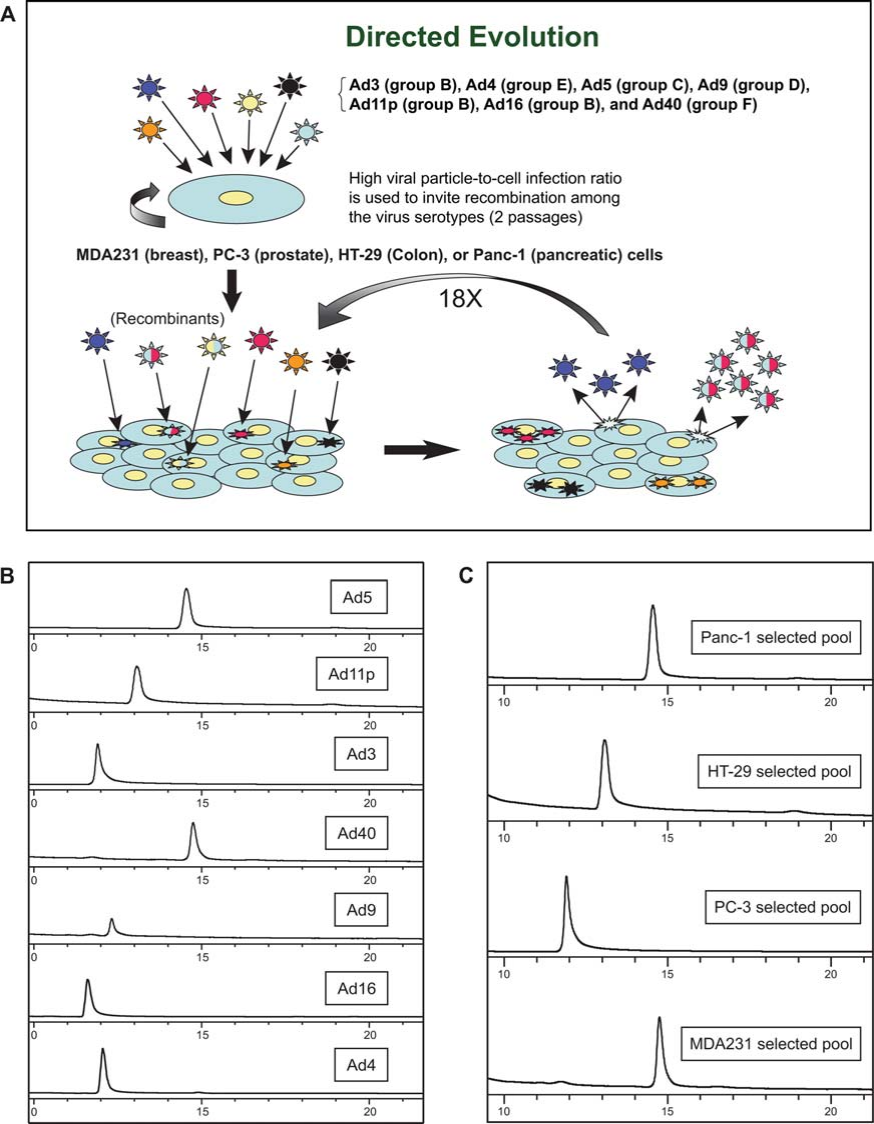
The Directed Evolution process and analysis of viruses and derivative viral pools by anion-exchange chromatography. *A,* Representation of the Directed Evolution process (see Materials and Methods for detailed description). *B,* Chromatograms of each pure Ad serotype included in the mixed serotype starting pool from which ColoAd1 was selected. *C,* Chromatograms of the passage 20 viral pools derived on the HT-29, Panc-1, MDA-231, and PC-3 tumor cell lines, respectively. The differing retention times of these pools are consistent with the predominant serotype of the pool being Ad5 or Ad40 for the Panc-1 pool, Ad11p for the HT-29 pool, Ad3 or Ad4 for the PC-3 pool, and Ad5 or Ad40 for the MDA-231 pool.

The Directed Evolution strategy outlined in the Materials and Methods (Figure 1*A*) is an unbiased approach to determine whether alternative serotypes, or recombinants thereof, are superior in potency to Ad5 (the serotype of all current oncolytic Ads) on human cancer cell lines. As a low-resolution method to track changes during passage of the viral pool and to characterize the homogenous/heterogenous nature of the final viral pool, the pools were examined on a TMAE anion exchange column exploiting viral capsid charge differences associated with each serotype (Figure 1*B*). Each viral pool collected from the different cell lines after passage 20 eluted as a single peak with distinct retention time (Figure 1*C*). Each elution peak of a passaged viral pool appeared to track with one of the original serotypes (Figure 1*B*). This suggests that all viruses are not equal in their potency on a given tumor cell line and that differences in tumor cell lines can select for specific viruses in a mixed virus pool. Since at least two of these selected viral pools did not track with the Ad5 retention time, Ad5 is not (based on potency) the best virus for deriving all oncolytic Ads.

An MTS assay was employed to compare the potency of the different selected viral pools to the original mixed serotype pool and to Ad5. All selected viral pools increased in potency relative to Ad5 or the starting pool, with the magnitude of the increase varying significantly between the pools. The greatest increase in potency relative to Ad5 was observed in the pool passaged on the colon tumor cell line HT-29 (approximately 2 log increase) with the smallest increase (1.2 fold) noted in the pool derived from passage on the MDA-231mt1 cell line (Table 1).

**Table 1:** Potency of viral pools relative to Ad5 on cognate cell lines.

Cell Line	Virus	IC_50_ (Vp/cell)	Potency (Relative to Ad5)
HT-29	Ad5	20	
	Wt0	20	1
	HT29(Wt20)	.03	667
PC-3	Ad5	40	
	Wt0	8.0	5
	PC-3(Wt20)	9.5	4.2
MDA231	Ad5	30	
	Wt0	80	.37
	MDA231(Wt20)	25	1.2
Panc-1	Ad5	9.5	
	Wt0	15	.07
	Panc-1(Wt20)	3.5	2.7

Potency values less than 1 indicates attenuation relative to Ad5.

The potency of each viral pool that underwent 20 passages of Directed Evolution (Wt20) was compared to the starting pool (Wt0) and to Ad5 on their respective cognate cell line. Viral potencies were measured by MTS assay.

### ColoAd1 is a highly potent and selective oncolytic virus

Since the viral pool passaged on HT-29 cells displayed the greatest increase in potency on its cognate cell line, viruses within this pool were pursued for further characterization. Individual plaque-purified viruses were isolated and screened by MTS assay for their lytic potential on the HT-29 tumor cell line. The potency of individual plaques was compared to that of the HT-29 pool from which they were isolated. The plaque-purified viruses were found to be equal to or greater in potency than the HT-29 pool. The most potent of these plaque-purified viruses, termed ColoAd1, was chosen for further characterization.

While ColoAd1 was selected for growth on the colon tumor cell line HT-29, it was not clear whether this virus had increased potency on all tumor cell lines, was selective for colon cancer tumor cell lines, or was more potent on all cell types including primary normal cells. To address the first two questions, ColoAd1 was tested by the MTS assay on all of the original tumor cell lines used in this study (Panc1-sct, MDA-231mt1, HT-29, and PC-3), two additional tumor cell lines (OVCAR-3, DU-145), and on a panel of colon tumor cell lines (DLD-1, LS1034, HCT116, LS174T, SW48, SW403, Colo320DM), comparing it to Ad5. ColoAd1 was over 2 logs more potent than Ad5 on the cognate cell line, HT-29. Additionally, ColoAd1 demonstrated potency equal to or greater than Ad5 on some human tumor cell lines (e.g. PC-3, MDA-231, Ovcar-3, and DU-145), but was attenuated (about two logs less potent than Ad5) on the Panc1 cell line (Table 2). ColoAd1 displayed significantly increased potency (9 to 100 fold) relative to Ad5 on all colon cancer tumor cell lines screened, with the exception of Colo320DM (Table 3). This suggests that colon tumor cell lines have properties that make them significantly susceptible to infection and lysis by ColoAd1.

**Table 2:** Potency of ColoAd1 relative to Ad5 on a panel of cancer cell lines.

Cell Line	Virus	IC_50_ (Vp/cell)	Potency (Relative to Ad5)
HT-29	Ad5	73	1282
	ColoAd1	0.06	
PC-3	Ad5	11	48
	ColoAd1	0.23	
MDA231 mt1	Ad5	17	3
	ColoAd1	5	
Ovcar-3	Ad5	18	1.5
	ColoAd1	12	
DU145	Ad5	1	1
	ColoAd1	0.84	
Panc-1	Ad5	0.05	0.02
	ColoAd1	3	

Potency values less than 1 indicates attenuation relative to Ad5.

Potency of ColoAd1 on a mixed panel of tumor cell lines. The potencies of ColoAd1 and Ad5 were measured by MTS on the mixed panel of tumor cell lines to derive an IC_50_ value for each virus. These IC_50_ values were used to derive the potency of ColoAd1 relative to Ad5 using the calculation IC_50_ value Ad5 divided by the IC_50_ value of ColoAd1 on the same cancer cell line.

**Table 3 pone-0002409-t003:** Potency of ColoAd1 relative to Ad5 on a panel of colon cancer cell lines.

Cell Line	Virus	IC_50_ (Vp/cell)	Potency (Relative to Ad5)
HT-29	Ad5	73	1282
	ColoAd1	0.06	
DLD-1	Ad5	35	100
	ColoAd1	0.35	
LS1034	Ad5	8	38
	ColoAd1	0.21	
HCT116	Ad5	0.72	36
	ColoAd1	0.02	
LS174T	Ad5	13	23
	ColoAd1	0.57	
SW48	Ad5	1	17
	ColoAd1	0.06	
SW403	Ad5	9	9
	ColoAd1	1	
Colo320DM	Ad5	3	0.03
	ColoAd1 *r2 value 0.82	105	

Potency values less than 1 indicate attenuation relative to Ad5.

Potencies of ColoAd1 and Ad5 were measured by MTS on the mixed panel of tumor cell lines to derive an IC_50_ value for each virus. These IC_50_ values were used to derive the potency of ColoAd1 relative to Ad5 using the calculation IC_50_ value Ad5 divided by the IC_50_ value of ColoAd1 on the same colon tumor cell line.

To test whether ColoAd1 was selective for tumor cells over normal cells and thus, by definition, an oncolytic virus, ColoAd1 was examined in two different colon tumor cell lines (HT-29, DLD-1), and on primary endothelial and epithelial cells (HUVEC, HMEC) comparing its potency in each MTS assay to Ad5 and ONYX-015/H101. The results show that ONYX-015 and Ad5 are significantly less potent than ColoAd1 on the HT-29 and DLD-1 cell lines (Table 4). In contrast, the potency of ColoAd1 on HUVEC cells was the same as Ad5 and slightly more potent than ONYX-015 (Table 4). On HMEC cells, ColoAd1 was less potent than Ad5 and ONYX-015 (Table 4). To quantitate these differences between ColoAd1, ONYX-015, and Ad5, an *in vitro* therapeutic window was calculated, defined as the ratio of the IC_50_ of a given virus on normal cells, HUVEC or HMEC, divided by the IC_50_ on the colon tumor cell lines HT-29 or DLD-1 (Table 2). These calculations establish that ColoAd1 has a therapeutic window that is 3 to 4 logs greater than that of Ad5 or ONYX-015/H101 in these *in vitro* assays.

**Table 4:** Potency and therapeutic indices of ColoAd1.

Cell Line	Virus	IC_50_ (Vp/cell)	Potency (Relative to Ad5)	Therapeutic Index (IC_50_ HUVEC/IC_50_ HT-29)	Therapeutic Index (IC_50_ HMEC/IC_50_ HT-29)
HUVEC	Ad5	36			
	Ad3	799	0.05		
	Onyx-015/H101	391	0.1		
	Ad11p	399	0.1		
	ColoAd1	50	0.7		
HMEC	Ad5	26			
	Ad3	43	0.6		
	Onyx-015/H101	9	3		
	Ad11p	940	.03		
	ColoAd1	575	.05		
HT-29	Ad5	11		3	2
	Ad3	45	0.2	18	1
	Onyx-015/H101	140	0.1	3	.06
	Ad11p	1.9	6	214	495
	ColoAd1	.02	650	2500	28750
DLD-1	Ad5	5		7	5
	Ad3	1162	0.004	0.7	.04
	Onyx-015/H101	461	0.01	0.8	.02
	Ad11p	4	1	100	235
	ColoAd1	.03	167	1667	19167

Potency values less than 1 indicates attenuation relative to Ad5.

The potencies of ColoAd1, Ad5, ONYX-015, Ad3 and Ad11p on colon cancer (HT-29, DLD-1) and normal cells (HUVEC, HMEC) were measured by MTS assay at day 4 post-infection and is represented in the table as an IC_50_ value. The potency (as reflected in the IC_50_) is the ratio of the IC_50_ of a given virus on a given cell line relative to Ad5's IC_50_ on that cell line. The Therapeutic Index was calculated using either HUVEC (primary endothelial) or HMEC (primary epithelial) cells and using the ratio of the IC_50_ of a virus on these primary, normal cells and dividing it by its IC_50_ on HT-29 tumor cells.

### ColoAd1 is a chimeric virus that displays enhanced potency over its parent virus, Ad11p

**Figure 2:**
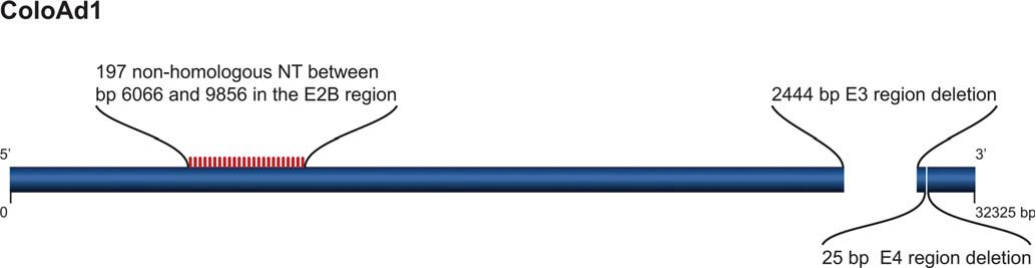
Genomic sequence diagram of ColoAd1. The genomic differences between ColoAd1 and Ad11p are noted in the schematic. In the E2B region there are frequent substitutions of Ad3 sequences for Ad11p sequences between base pairs 6081 and 9322. In addition, ColoAd1 has a nearly complete (2,444 bp) E3 region deletion, and a smaller (25 bp), second deletion that maps to a putative E4orf4 region of the virus.

Chromatographic analysis indicated that the major coat proteins of ColoAd1 were derived from Ad11p, a group B virus. To determine the relationship between ColoAd1 and Ad11p, the virus was sequenced, revealing that ColoAd1 is Ad11p, with a nearly complete E3 region deletion, a smaller deletion in the E4 region, and a chimeric Ad3/Ad11p E2B region (Figure 2).

It is possible that Ad11p or Ad3 are serotypes that are inherently more potent, and have a wider therapeutic window, than Ad5, and that this property is independent of the acquired genetic changes found in the ColoAd1 genome. To test this, we examined ColoAd1, Ad11p and Ad3 on the two colon tumor cell lines, HT-29 and DLD-1, and on primary human endothelial and primary human epithelial cells, HUVEC and HMEC, respectively, by MTS assay. As presented in Table 4, ColoAd1 exhibited superior potency over both Ad11p and Ad3 on the colon tumor cell lines, demonstrating that this recombinant virus was superior in potency to both of its parent viruses. Interestingly, Ad11p, and not Ad3, displayed an inherent therapeutic window as defined here via MTS analysis. Thus, ColoAd1 is a derivative of Ad11p whose differences with Ad11p enhance the potency of the virus without altering the serotype's natural ability to selectively replicate in tumor cells versus primary normal cells.

It is important to note that the reported seroprevalence of Ad11p is low [22], [23] The greatest need for this type of therapeutic is in patient populations where the tumor has progressed to a systemic, metastatic cancer. Thus treating patients with an agent that lacks pre-existing immunity should enhance the opportunity for the agent to circulate and eliminate metastatic tumor cells. To confirm the previous reports of low seroprevalence of Ad11p, serum from six different individuals was collected and tested for the ability to neutralize the infectivity and lytic potential of these viruses on a reporter cell line, Ovcar-3. In agreement with the literature, the serum demonstrated little effect on ColoAd1 (data not shown), suggesting that ColoAd1 may be a viable approach for the systemic treatment of colon cancer.

### ColoAd1 has anti-tumor activity superior to ONYX-015 and Ad11p in a colon cancer liver tumor seeding xenograft mouse model following i.v. administration

**Figure 3:**
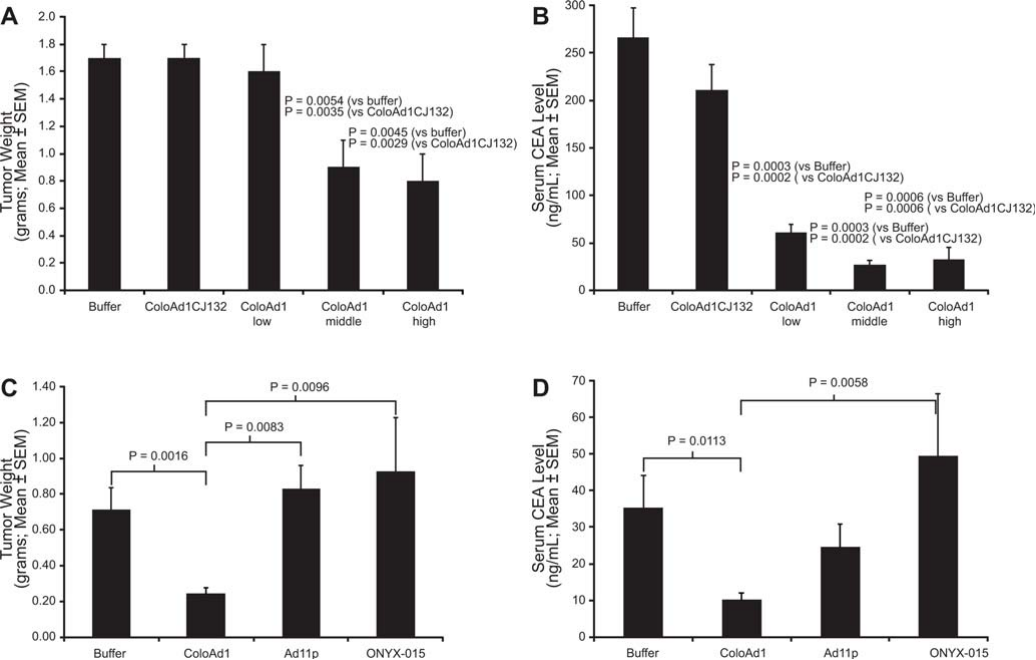
Anti-tumoral activity of ColoAd1 after systemic administration in a liver metastasis xenograft mouse model. HT-29 colon cancer cells were seeded to the liver of nude beige mice (n = 10 mice per treatment group). Plasma CEA level was used to monitor tumor establishment. *A* and *B*, Mice were treated by tail-vein (i.v.) injection with 1×10^10^, 5×10^10^, or 1×10^11^ total viral particles of ColoAd1 per mouse. A fourth set of liver-tumor-bearing mice were i.v. injected with 1×10^11^ total viral particles of a replication-defective [E1^(−)^] version of ColoAd1, ColoAd1CJ132. A fifth set of mice were injected with vehicle control (buffer). Tumor weight measurements demonstrate that ColoAd1 has anti-tumoral activity, which is dose-dependent (*A*). Blood CEA levels at the end of the study (day 12 post viral administration) corroborate the tumor weight data (*B*). *C and D,* Comparison of anti-tumoral activity of ColoAd1, Ad11p and ONYX-015 in the HT-29 liver metastasis xenograft mouse model. In a second study performed in the same model as in Panels A and B, ColoAd1 was compared to its parental virus, Ad11p, and to the clinically-approved oncolytic virus ONYX-015; each virus dosed i.v. to a total of 1×10^11^ viral particles per mouse.

Most solid tumors are metastatic at the time of diagnosis. Since the initial site of metastasis for colon cancer is the liver, a colon cancer liver tumor seeding model [24] was used to examine the *in vivo* efficacy of ColoAd1. To determine first whether viral anti-tumoral activity is dependent upon the ability of the virus to replicate and spread in this model, a dose response study was conducted comparing ColoAd1 to a replication-defective form of ColoAd1 (where the essential E1 region of the virus was deleted). As seen in Figure 3*A*, systemically delivered ColoAd1 significantly decreases tumor burden in a dose, and replication, dependent fashion. Since HT-29 cells shed carcinogenic embryonic antigen (CEA), this can be used as an easily measured surrogate for tumor burden. Importantly, measurements of CEA in the bloodstream (Figure 3*B*) correlated well with the results of tumor-weight measurements (Figure 3*A*).

To test whether the superior *in vitro* potency of ColoAd1 relative to Ad11p and ONYX-015/H101 was recapitulated *in vivo*, these viruses were compared in the liver tumor seeding model. As seen in Figure 3*C* (tumor weight) and Figure 3*D* (CEA level measurements), the anti-tumoral activity of ColoAd1 was superior to both Ad11p and ONYX-015 in this model, corroborating the *in vitro* conclusions.

### Selectivity of ColoAd1 on human tumor tissues isolated from colon cancer patients

**Figure 4:**
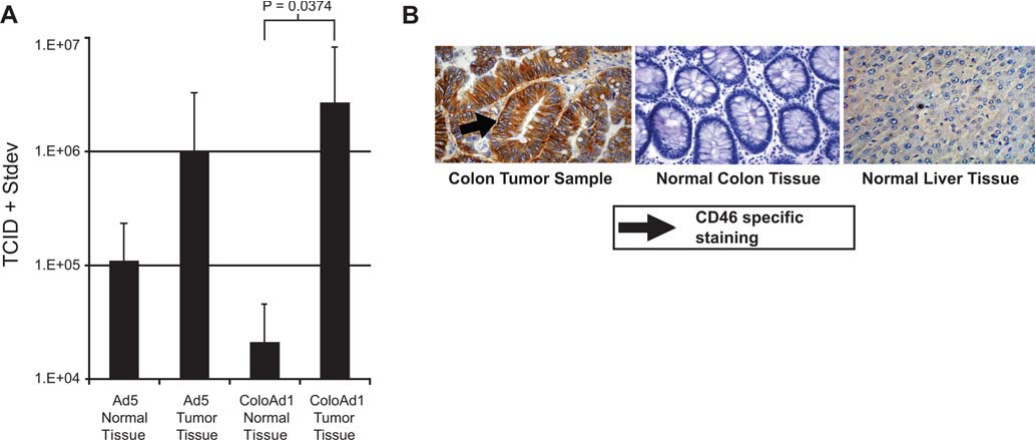
*Ex vivo* potency and selectivity of ColoAd1. *A,* Punch samples of freshly-excised human colon tumors (n = 6) and matched normal margin areas, were infected with either ColoAd1 or Ad5 and maintained in tissue culture. The viral burst from each sample was measured by plaque assay at 24 hours post infection. *B,* Immunohistochemical staining for CD46 present in clinical colon tumor, normal colon, and normal liver samples.

*In vitro* and *in vivo* models that accurately predict clinical efficacy have been difficult to identify, and the lack of such prognostic models continues to result in extensive attrition of cancer drugs. Consequently, freshly isolated, surgically excised human colon cancer material was examined as an additional model system for testing ColoAd1. Since surgical material includes both tumor tissue and normal cell margins, this system offers an excellent opportunity to test the tumor selectivity of the virus in the context of intact human tissue. The viability of a series of tumor samples was analyzed in tissue culture; viability varied from 2 to 6 days. Consequently, to ensure that cell death was due to virally induced lysis and release of progeny virus and was not due to spontaneous lysis of the surgical material, a 24 hr endpoint was selected. Six tumor samples were collected and punch cultures from tumor and normal sections (as determined by a clinical pathologist) were generated and exposed to either Ad5 or ColoAd1. To determine each viruses' ability to replicate, lyse and release infectious virus, supernatant was collected 24 hr post-infection and assayed for the presence of progeny virus. As seen in Figure 4*A*, ColoAd1 generated approximately two logs more viral progeny on tumor material than on matched normal tissue, confirming its tumor selective replication in freshly isolated human tumor tissue. These studies also demonstrated that ColoAd1 exhibited at least one log higher tumor selectivity than the control virus, Ad5.

CD46 has been identified as a cellular attachment receptor for Ad11p, the parent virus of ColoAd1 [25], [26]. To better define the expression of CD46 in primary and metastatic colon cancer, we examined colon cancer material, normal liver tissue and normal colon tissue for CD46 expression by immunohistochemistry (IHC) (Figure 4*B*). Strong CD46 IHC staining was consistently seen in colon cancer tissue, but was absent or generally weak in normal colon and liver tissue. This suggests that CD46 expression may be a contributing factor to the observed tumor selectivity of ColoAd1 and thus may be a potential tool for pre-screening patients for treatment with this therapeutic agent.

### ColoAd1 can be armed without compromising potency

**Figure 5:**
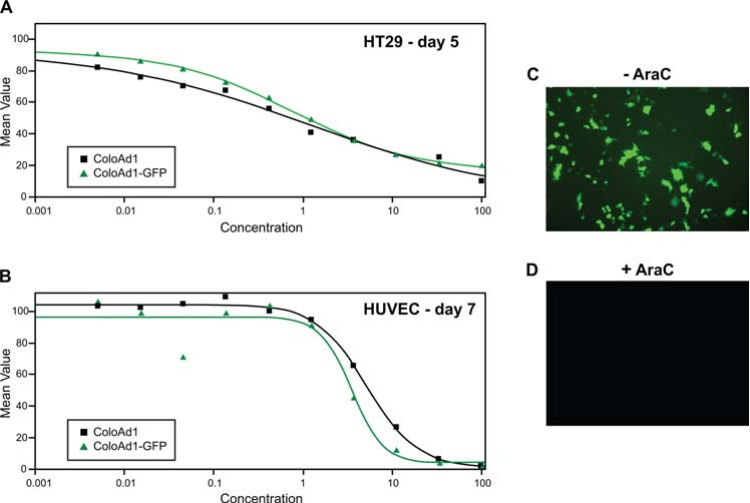
Potency and kinetics of armed virus, ColoAd1-GFP. MTS assays were performed on ColoAd1 and ColoAd1-GFP on A) the colon tumor cell line, HT-29 and B) the primary endothelial cells, HUVEC. The reporter gene, GFP, is expressed with late kinetics (ie., is dependent upon the initiation of viral DNA replication for expression) as defined by expression C) only in the absence of AraC and D) lack of expression in the presence of AraC.

Armed oncolytic viruses seek to complement the potency of the oncolytic virus by the addition of therapeutic transgenes [20]. In this approach it is important that a therapeutic transgene insertion site within the viral genome be identified that does not compromise the life cycle and therefore the potency of the virus. Unlike Ad5, where the biology and description of insertion sites compatible with the viral life-cycle are well described, ColoAd1 represents a novel agent that is primarily derived from the poorly studied Ad11p genome. Consequently, a transposon-based system that can scan the genome for insertion sites in a non-prejudiced fashion was utilized for the identification of compatible transgene insertion sites [27] Given that the viral genome coding capacity of the human Ad is constrained [28] a consensus splice acceptor site was placed upstream of the transgene, eliminating the need for an exogenous promoter and linking expression to an endogenous ColoAd1 promoter [29]. To enhance the ability to identify transgene expressing ColoAd1 variants, GFP was chosen as the transgene. A number of viral isolates were generated and then screened for potency and a virus termed ColoAd1-GFP was selected based on equivalent potency to the parent ColoAd1 (Fig. 5A and 5B).

Past studies using a splice acceptor-based expression cassette demonstrated that expression occurred late in the viral life cycle and was dependent upon viral DNA replication [29]. Linking therapeutic transgene expression to the selectivity of the virus has a significant safety advantage over traditional constitutive expression systems since gene expression would be limited and dependent upon the tumor selectivity of the viral system [30]. To determine the GFP expression kinetics from ColoAd1-GFP, HT-29 cells were infected in the presence or absence of AraC, a compound which inhibits viral replication. As seen in Figures 5C and 5D, the GFP expression was blocked by the addition of AraC indicating that expression occurs late in the viral life cycle and is linked to viral replication.

## Discussion

In the present study we established conditions that select potent viral agents, without bias towards any mechanism, from a pool of Ad serotypes representing Ad subgroups B–F. This method, which is a highly accelerated version of the natural selection of viruses, can be applied to any virus and any cancer type of choice.

Using this process, we generated and characterized ColoAd1, a novel Ad3/Ad11p chimeric oncolytic virus for the treatment of human colon cancer and, potentially, other indications. This virus was shown to be more potent and have a larger therapeutic window than Ad5 and the most clinically advanced oncolytic virus Onyx-015 (Tables 2–4). Futhermore, ColoAd1 demonstrated increased potentcy in an intravenous tumor model and on tumor explants (Figures 3 and 4).This virus has several changes relative to the parent Ad11p virus, including a chimeric E2B region and deletions in the E3 and E4 regions. Which change or changes play a role in the enhanced potency of this virus is not clear. The loss of genes in the Ad5 E3 region has been shown in group B Ads to enhance viral lysis and spread [31] by an undefined mechanism. The E2B region encodes the pre-terminal protein (pTP) and the viral DNA polymerase (DNA pol), two of the three E2-encoded proteins necessary for viral DNA replication. The terminal 18 bp of the viral genome, considered the minimal replication origin, directly interacts with the pTP and DNA pol heterodimer. It is important to note that when sequenced the genomic ends of ColoAd1 and the wild type Ad11p were identical to those of Ad3 and conflicted with the described DNA sequence termini described for Ad11p [32], [33]. Thus, the E2B alterations in ColoAd1 may generate a pTP-DNA pol heterodimer that is more compatible with the terminal 18bp of ColoAd1 than the original Ad11p pTP-DNA pol. Coupled with ColoAd1's smaller genome (a result of genomic deletions) this virus may replicate more quickly and also reach a critical viral burst size more rapidly, consequently enhancing viral lysis and spread.

With regard to selectivity, studies with the pTP of Ad5 have shown that it interacts with CAD, a host protein responsible for the TP-nuclear matrix association. The level of CAD is correlated with the rate of cell division; two to five-fold higher levels in tumor cells than in normal cells and almost non-existent in quiescent cells [34], [35]. Consequently, TP alterations and their potential interactions with CAD (or similar proteins) may also be mechanisms of enhanced replication and/or selectivity of the virus.

The third alteration in the ColoAd1 viral genome is a small deletion (24 bp) that maps to the E4orf4 region of the virus. The E4orf4 protein of Ad5 interacts with the host cell's serine/threonine protein phosphatase 2A (PP2A), [36]. This interaction has been shown to induce p53-independent apoptosis, inactivate splicing factors, and reduce E1A activation of AP-1, JunB, and expression of the Ad E2 and E4 transcription units [37]. However, since the E4 region is highly spliced, the E4orf4 gene deletion may also alter the expression of another E4 gene in this complex transcription unit, thus indirectly contributing to the enhanced potency of ColoAd1. In addition, it is not clear that Ad11p and Ad3 proteins maintain any or all of the functions ascribed to homologous Ad5 proteins. Thus extrapolations of Ad5 protein functions to proteins in ColoAd1 must be carefully tested. Consequently, while it is clear that CololAd1 has undergone a series of genetic alterations that result in a more potent virus, it is not clear which alteration(s) are responsible for enhanced potency.

It is important to note that ColoAd1 is a member of the group B Ads and thus distinctly different from the traditional Ad5-based oncolytic viruses. It does not, for example, use the Ad5 receptor, (coxsackie B- and adenovirus receptor, CAR), for attachment to cells. Instead, ColoAd1 appears to employ at least two receptors that are distinctly different from CAR [22], [38], [39], one of which has been recently described as CD46 [25], [26]. The significance of this is emphasized by recent studies on clinical material showing that CAR is poorly expressed on a variety of different tumor types and that CAR expression decreases with the advance in stage and grade of the tumor [40], [41], [42], [43], [44]. Additional reports of tumor suppressor properties of CAR [45], [46] and detection of soluble CAR in the tumor microenvironment [47] call into question the use of CAR-dependent adenoviruses for the treatment of all human cancers. In contrast, tumor cell surface expression of CD46, the putative ColoAd1 receptor, appears to increase with stage and grade in a variety of cancers [48]. Thus, ColoAd1 may have therapeutic utility beyond colon cancer, and studies to investigate this are ongoing. Of additional importance are data demonstrating that the seroprevalence of Ad11p is low [22], [23]. Since the greatest need for this type of therapeutic is in patient populations where the tumor has progressed from a confined local disease to a systemic, metastatic cancer, treating patients with an agent to which they do not have pre-existing immunity should enhance the opportunity for the agent to circulate and eliminate metastatic tumor cells. This is in contrast to Ad5 where sero-prevalence, as measured by neutralizing antibodies, reaches levels of approximately 50% in the general population [23], [49].

It is important to note that the potency of ColoAd1 can be complemented by one and potentially more therapeutic transgenes. The ability to arm these agents represents a unique opportunity to impact the treatment of cancer on multiple levels from a single agent. As demonstrated by the incorporation and efficient expression of GFP from the ColoAd1 genome, arming can occur without compromising the potency or selectivity of the viral therapeutic. In addition to incorporating agents that complement the oncolytic potential of the virus (e.g. prodrug converting enzymes, anti-angiogenic factors, immunotherapeutics,), arming creates the opportunity for clinicians to track the activity of the virotherapy treatment in a minimally invasive fashion [50]. This is made all the more meaningful if the method for tracking the virotherapy is directly linked to the viral life-cycle. In the case of ColoAd-GFP, this has clearly been demonstrated, where GFP expression was demonstrated to be directly linked to DNA replication (Figure 5B). Various genes have been identified that would allow clinicians to track viral activity and include genes associated with radionuclide imaging (e.g. HSV-1 TK, human thyroidal sodium iodide symporter,[51], [52] ) and soluble marker peptides readily detectable in the bloodstream or via urine sampling (e.g. human carcinoembryonic antigen, β-chain of human chorionic gonadotropin [53], [54], [55]). Using the expression of the linked gene as a biomarker of viral replication and spread represents an opportunity for clinicians to personalize the treatment, giving additional doses only as needed, thus moving away from standard, timed dosages commonly associated with current chemotherapy treatments. Equally important, as we consider balancing the need for increased potency with safety of the viral therapy, arming could also be used to incorporate a “safety valve” into the virotherapy, capable of aborting the viral-based therapy through the administration of a clinically approved drug (eg. incorporation of the HSV TK gene and administration of gancyclovir). Importantly, the capacity to arm an oncolytic virus creates an opportunity to build increased potency, safety or enable more personalized medicine, a flexibility unique to this type of anti-cancer agent.

Human oncolytic viruses to date have failed in the clinic due to insufficient therapeutic efficacy as monotherapies. To address this, we expanded our search beyond the traditional Ad5 serotype to a series of Ad serotypes representing different viral subgroups. Inviting recombination to increase biodiversity, then applying selective pressure, we developed a unique oncolytic virus, ColoAd1. Deriving oncolytic viruses via tumor selection from serotype pools exploits the complex biology of both the viral agent and the tumor. While we demonstrate the principle using adenoviruses, the same approach may be applicable to other viruses and represents a novel approach to develop more effective virotherapies for the treatment of human tumors.

## Methods

### Viruses and Cell lines

The Ad serotypes Ad3 (GB strain), Ad4 (RI-67 strain), Ad5 (Adenoid 75 strain), Ad9 (Hicks strain), Ad16 (Ch. 79 strain) and the tumor cell lines A549, PC-3, HT-29, DLD-1, LS1034, HCT116, LS174T, SW48, SW403, Colo320DM, OVCAR-3, DU-145 were all purchased from the ATCC. HEK293s were licensed from McMaster Univeristy. MDA-231mt1 and Panc-sct were derived from rapidly growing subcutaneously implanted xenograft by Drs. Deb Zajchowski and Sandra Biroc at Berlex Biosciences, respectively. Human umbilical vein endothelial cells (HUVEC, Vec Technologies, Rensselaer, NY), and human mammary epithelial cells (HMEC, Cambrex, Walkersville, MD) were grown as per vendors instructions and Ad11p (Slobitski strain), and Ad40 were kind gifts from Dr. William S.M. Wold at St. Louis University. The replication defective ColoAd1 (bp 461–3397 deleted so as to eliminate the E1A and E1B genes) was derived by homologous recombination of ColoAd1 into a pBR-derived plasmid in BJ5183 bacteria using methods as previously described [56].

### Viral Purification and Quantitation

Viral stocks were propagated on HEK293 cells, with the exception of the replication-defective ColoAd1 which was propagated on A549 cells expressing the E1A and E1B regions of ColoAd1, and purified on CsCl gradients [57], [58]. The method used to quantitate and partially characterize viral particles is based on that of Shabram et al [59], with the exception that the anion-exchange media TMAE Fractogel was used instead of Resource Q [60].

### Cytolytic assay

The viral lytic capacity was measured using a modification of the MTT assay [61]. Briefly, the MTS assay (CellTiter 96® Aqueous Non-Radioactive Cell Proliferation Assay, Promega, Madison, WI) was used in place of the MTT assay because conversion of MTS by cells into aqueous, soluble formazan reduces time and eliminates the use of a volatile organic solvent associated with the MTT assay.

To perform the assay, cells were seeded at a density determined for each tumor cell line to generate a confluent monolayer within 24 hr. These densely seeded cells were allowed to grow for two additional days prior to exposure to the test virus(es). Infections of both tumor and primary normal cells were carried out in quadruplicate, using serial three fold dilutions of the viruses starting at a particle per cell ratio of 100 and ending at a particle per cell ratio of 0.005 with the exception of MTS assays on HUVEC or HMEC cells which were done starting at a particle per cell ratio of 10,000 for purposes of calculating an *in vitro* therapeutic index. Infected cells were incubated at 37°C and the MTS assay was performed at the time points indicated for the individual primary cells or tumor cell lines. Mock-infected cells served as negative controls and established the 100% survival point for the given assay.

### Directed Evolution and generation of ColoAd1GFP

Viral serotypes representing Ad subgroups B–F were pooled and passaged twice on sub-confluent cultures of the target tumor cell lines at a particle-per-cell ratio of approximately 200 to invite recombination between serotypes (Figure 1*A*). Supernatants from the second round of the high viral particle-per-cell infection of subconfluent cultures were then used in a 10-fold dilution series to infect confluent T-75 tissue culture flasks of target tumor cell lines PC-3, HT-29, Panc-1 and MDA-231. To achieve confluency, each cell line was seeded at split ratios that allowed that cell line to reach confluency between 24 and 40 hours post seeding, and the cells were allowed to grow a total of 72 hours post seeding prior to infection. This was done to maximize the confluency of the cells attempting to mimic growth conditions in human solid tumors.

The infected T75s were observed for the first signs of cytopathic effect (CPE). In order to harvest the most potent viruses, cell culture supernatant was harvested from the flask infected with the most concentrated innocula in the 10-fold dilution series that did not show any sign of CPE at day 3 or 4 post-infection. The assumption was that only a small population of potent viruses would be generated and these viruses would replicate, lyse the host cells and be released into the supernatant before any gross morphological changes could be detected. In the case of HT-29 and PC-3 cell lines, this was modified for passages 10–20 to harvest of the second flask, i.e. harvest 100-fold below the dilution in which CPE were detectable by day 3 post-infection. Each harvest served as the starting material for the successive passage of the virus. This process was repeated until the viral pool achieved 20 passages.

Individual viruses were isolated from each passage 20 pool by two rounds of plaque purification on A549 cells using standard methods [57]. In brief, dilutions of the supernatant harvested from the 20^th^ passage on each target tumor line were used to infect A549 cells in a standard plaque assay. Individual plaques were harvested, and the same plaque assay method was used to generate a second round of individual plaques from these harvests. Plaques from the second round of plaque purification were deemed pure, infected cultures were prepared using these purified plaques, and the potency of these culture supernatants determined by MTS assay as described.

ColoAd1GFP was generated using a transposon-based arming system as previously described [29]. Briefly, ColoAd1 genomic DNA was isolated and cloned into a pBR-derived plasmid by homologous recombination in BJ5183 bacteria to create plasmid pCJ94. In pCJ94, the viral genome is flanked on both sides with AsiSI restriction enzyme sites to allow the viral genome to be excised from the plasmid back and thus enable efficient virus rescue. Using transposition, an expression cassette containing a consensus splice acceptor upstream of the green fluorescent protein (GFP) gene was inserted at random sites throughout the ColoAd1 genome within the pCJ94 plasmid. The recombinant ColoAd1-GFP genomes where then isolated from a plasmid pool by AsiSI restriction enzyme digestion and transfected into HEK-293 cells [27]. Using a fluorescent microscope, GFP positive plaques were picked and propogated in A549 cells. Three rounds of plaque purification were performed. CPE stocks of 4 recombinant viruses were generated and titered by HPLC [60]. Of these 4 recombinant viruses a single clone, termed ColoAd1-GFPdemonstrated potency equivalent to ColoAd1 as determined by MTS assay on HT-29 and HUVEC cells as described. Clone 4cli2a was chosen for further study and termed ColoAd1-GFP. Viral DNA replication dependent GFP expression from ColoAd1-GFP was determined by assaying expression in the presence of the DNA replication inhibitor AraC (Sigma Aldrich, St. Louis, MO).

### DNA sequencing

Purified ColoAd1 and Ad11p DNA samples were sent to Commonwealth Biotechnologies Inc. (CBI, Richmond, VA) for sequencing. The DNA was partially digested with the restriction endonuclease Sau3 AI, and “shotgun” cloned into the plasmid vector pBluescript II. Positive clones were propagated, the plasmid isolated and sequenced using the sequencing primers M13R and KS. Individual sequencing reactions were trimmed, edited and assembled using Sequencher™ (Gene Codes Corp.). Gaps in coverage were amplified with custom oligonucleotide primers and sequenced. The 5′ and 3′ ends were sequenced directly off the ColoAd1 and Ad11p DNA (1 µg DNA per reaction).

### *In vivo* and *ex vivo* testing

Mouse studies were performed as previously described [24] in accordance with the institutional guidelines of the University of Washington. All experiments involving animals were conducted in accordance with the institutional guidelines set forth by the University of Washington. Briefly, 8- to 12-week-old female immunodeficient mice (CB-17/lcrCrl-scid-bgBR; CB-17; Charles River Laboratories Inc., Wilmington, MA.) were housed in specific pathogen-free facilities and infused with 2×10^6^ HT-29 cells (human colorectal adenocarcinoma, ATCC# HTB-38) through a permanently placed portal vein catheter. Fifteen days after tumor cell transplantation, blood samples (80 ul) were obtained by retrorbital bleeding and serum was obtained and stored at −80°C for subsequent analyses. Preliminary studies determined that (tumor-derived) serum human CEA levels become detectable at day 15 after HT-29 cell transplantation. At day 16 and 17 post tumor transplantation, mice received the indicated doses of viruses (in 200 ul PBS) by tail vein injection. Control mice were injected with 200 ul of PBS. At days 4, 8, 12, and 15 after virus injection, mice were weighed and blood samples were collected. At day 15 after virus injection, mice were sacrificed, tumor-bearing livers were micro-dissected and weighed and tumor burden was expressed as the ratio of tumor per total liver weight. Serum CEA levels were measured by ELISA (Calbiotech, Spring Valley, CA) according to the manufacturer's manual.

For clinical tissue biopsy, *ex vivo* culture and viral infectivity analysis, an *ex vivo* culture system, using clinically derived tumor or normal tissue biopsies, was developed. Tissue specimens were collected and processed at the Veterans Affairs Palo Alto Hospital (Palo Alto, CA) under the institutional review board-approved procedure. All the clinical tissues were obtained with approval of the research ethics committees and with informed consent. The trial was carried out under the oversight of the Institutional Review Boards of Stanford University and the Palo Alto Veteran's Administration Health Care System. Written consent was obtained for use of the tumor samples. Immediately after surgical removal the tissue specimens were dissected on ice and homogeneous areas of tumor and non-tumor regions were identified by a pathologist. By dicing the specimens into crossed surgical blocks, cubes of less than 1 mm^3^ were prepared, rinsed and placed in 6-well plates in Iscove's modified Dulbecco's medium (IMDM) supplemented with 5% FCS. Viral tissue infection was then achieved by addition of 1×10^10^ particles to each tissue sample. Virus was removed after 2 h, tissue washed and fresh medium added to the wells (medium as before, but with 10 µM insulin and 1 µM hydrocortisone). The tissue specimens were placed on Millicell 0.45 µm membrane culture inserts (Millipore, MA, USA) inside 6-well plates and incubated at 37°C, 5% CO_2_ for the duration of the study.

After 24 h the media was removed for titering of PFU and the tissue paraffin embedded. Evaluation of tissue survival, cytopathic effect and general tissue/cell morphology was performed by examination of Haematoxylin and Eosin (H/E) stained paraffin sections. Sections were stained for CD46 expression with mouse anti-human CD46 (RDI, Concord, MA) followed by anti-mouse biotin-conjugated secondary antibody and developed with a streptavidin-HRP complex and counterstained with Haematoxylin.

### Statistical Analysis

In vivo values were expressed as mean+/−standard error of the mean (SEM). Ex vivo data were expressed as mean+/−standard deviation (stdev). Differences between groups were analyzed by Mann-Whitney analysis. Values of *p*<0.05 were considered statistically significant. MTS assays were assessed in quadruplicate, and IC_50_ values were derived from dose response curves with R^2^ value of 0.9 or greater. *P* values were calculated by Mann-Whittney U-test. A *P* value of <0.05 was considered statistically significant. Each MTS assay was repeated at least 2–3 times, with consistent results.
